# The tuberculosis challenge in a rural South African HIV programme

**DOI:** 10.1186/1471-2334-10-23

**Published:** 2010-02-10

**Authors:** Catherine F Houlihan, Portia C Mutevedzi, Richard J Lessells, Graham S Cooke, Frank C Tanser, Marie-Louise Newell

**Affiliations:** 1 Africa Centre for Health and Population Studies, University of KwaZulu-Natal, Mtubatuba, South Africa; 2 Department of Infectious Diseases, Imperial College London, London, UK; 3 Centre for Paediatric Epidemiology and Biostatistics, University College London Institute of Child Health, UK

## Abstract

**Background:**

South Africa remains the country with the greatest burden of HIV-infected individuals and the second highest estimated TB incidence per capita worldwide. Within South Africa, KwaZulu-Natal has one of the highest rates of TB incidence and an emerging epidemic of drug-resistant tuberculosis.

**Methods:**

Review of records of consecutive HIV-infected people initiated onto ART between 1^st ^January 2005 and 31^st ^March 2006. Patients were screened for TB at initiation and incident episodes recorded. CD4 counts, viral loads and follow-up status were recorded; data was censored on 5th August 2008. Geographic cluster analysis was performed using spatial scanning.

**Results:**

801 patients were initiated. TB prevalence was 25.3%, associated with lower CD4 (AHR 2.61 p = 0.01 for CD4 <50 cells/μl) and prior TB (AHR 1.58 p = 0.02). Incidence was 6.89 per 100 person-years from 81 cases over 1175 person-years analysis time and was highest in the first 3 months after ART initiation; associated with male sex and higher log HIV RNA. Prevalent and incident TB were significantly associated with mortality (OR 1.81 p = 0.01 and 2.02 p = 0.01 respectively). Incident TB was associated with a non-significant trend towards viral load >25 copies/ml (OR 1.75 p = 0.11). A low-risk cluster for incident TB was identified for patients living near the local hospital in the geospatial analysis.

**Conclusion:**

There is a large burden of TB in this population. Rate of incident TB stabilises at a rate higher than that of the overall population. These data highlight the need for greater research on strategies for active case finding in rural settings and the need to focus on strengthening primary health care.

## Background

South Africa remains the country with the greatest burden of HIV positive individuals [1] and the second highest estimated TB incidence per capita worldwide [2]. Within South Africa, the province of KwaZulu-Natal has the greatest number of HIV positive individuals (an estimated 1.2 million people), with a sustained high incidence of new adult HIV infections [3]. TB notification rates are high with 1094 cases per 100,000 population, compared to a national incidence of 739.6 [4] and an emerging epidemic of drug resistant TB, which has received international attention [5]. Drug resistant TB has been identified as a major threat to the success of antiretroviral roll-out [6] and in areas where HIV and TB prevalence are high, both will require locally tailored interventions far beyond any current efforts. To add to limited data from urban and peri-urban settings [7,8], we set out to explore the burden and impact of TB in a cohort of patients initiating ART in rural KwaZulu-Natal. We further used novel geospatial clustering analysis to define areas where incident TB rates are higher or lower than might be expected; such areas could be used for targeted behavioural analysis, to aid our understanding of the TB epidemic in HIV infected individuals, and measure the impact of interventions to curb it.

## Methods

The study was carried out within the public sector ART programme of Hlabisa sub-district, Umkhanyakude, KwaZulu-Natal. The sub-district is predominantly rural with a population of approximately 228,000 people [9].

The Department of Health (DoH) HIV Treatment and Care Programme started in 2004 and is run in partnership with the Africa Centre for Health and Population Studies, with funding from PEPFAR http://www.africacentre.ac.za. HIV and TB services are decentralised to primary care, offering initiation of ART at 15 clinics by 2006. In 2004, the HIV prevalence was high at 22% overall, and peaked at 51% in females in 25-29 year age group [10]. Standard South African DoH approved ART regimens are used [11] (stavudine and lamivudine with either efavirenz or nevirapine in the initial regimen), with standard criteria for ART eligibility (stage 4 disease or CD4 <200 cells/μL). Once on treatment, patients are advised to return for CD4 and HIV RNA viral load testing six monthly and medication collection monthly.

During the preparation to start ART, patients were screened for TB, using symptom screen and AFB smear. In 2005, chest X-ray (CXR) was available only in the hospital, from early 2007, CXR was also available free in the local town. TB diagnosis and treatment were according to the National TB Control Programme Guidelines [12]. Sputum culture was not routinely available.

CD4 count, HIV RNA viral load, WHO clinical stage at baseline, date of initiation, ART regimen, TB treatment (at ART initiation and subsequently) and outcome (loss to follow-up, transfer out and death) were routinely collected in a database. Patient records were reviewed by a clinician to independently validate the data and record additional information, therefore only those with files available in the clinics were included (clinics store files of those lost to follow-up and deceased).

Ethics approval was granted by University of KwaZulu-Natal Biomedical Research Ethics board (Reference BE066/07) and the Research Office of the KwaZulu-Natal Department of Health.

### Statistical Analysis

Multivariate logistic regression analysis was used to examine baseline factors independently associated with prevalent TB. WHO stage at initiation was dropped in both univariate and multivariate analysis due to collinearity as all people with prevalent TB were WHO stage 3 or 4 by definition [11]. Incident rates of TB were calculated using Kaplan-Meier survival analysis. Cox proportional hazards regression modelling was used to evaluate associations with incident TB; person-time was censored on 5^th ^August 2008. Incidence rates were calculated as the total number of newly diagnosed TB cases divided by the time at risk of TB and also as the number of new TB cases divided by the person-time contributed before the first TB episode. Analysis time was censored at the time of death, last clinic visit (for lost to follow-up) or date of incident TB [13]. Independent variables were included in multivariate models if univariably associated with outcome at 15% significance level or if they affected associations involving other covariates. Patients with prevalent and/or incident TB were compared with those without to assess the effect of TB on mortality and virological failure. All statistical analyses were conducted using STATA version 10.0 (SAS institute, Cary, North Carolina, USA).

### Spatial Clustering analysis

A spatial scan was used to explore geographical differences in TB incidence across the sub-district. The Africa Centre maintains a GIS database of all local areas (a Zulu neighbourhood with a median population of about 400 people) in the sub-district. Patients without prevalent TB, residing within the sub-district and with a valid address, were carefully geo-coded to the centroid of their corresponding local area using our geographic database. We then applied a Kulldorff spatial scan statistic (implemented within the SaTScan spatial cluster detection programme [14]) to identify clusters of incident TB infections (either high or low numbers of infections) at a local area level using an exponential survival model [15]. A spatial scan statistic is a cluster detection test that is able to both detect the location of clusters and evaluate their statistical significance without problems associated with multiple testing. The statistical theory behind the spatial scan statistic is described in detail elsewhere [16].

The spatial scan statistic imposes a circular window on a map and it allows the centre of the circle to move across the study region. For any given position of the centre, the radius of the circle changes continuously so that it can take any value from zero up to a specified maximum value. The statistic evaluates the incidence of TB infection within a circle relative to expectation (based on the mean TB incidence for patients used in this analysis). The circle with the maximum likelihood is defined as the most likely cluster, implying that it is least likely to have occurred by chance. The p-value of the statistic is obtained through Monte Carlo hypothesis testing (9,999 iterations), where the null hypothesis of no clustering is rejected if the simulated p-value is < 0.05.

### Definitions

An episode of TB included any individual who started TB treatment irrespective of smear or culture positivity, similarly it encompassed any notified case of TB in the cohort. Prior TB was defined as any recorded episode of TB treatment, complete by time of ART initiation. Prevalent TB was defined as patients receiving TB treatment at ART initiation, including those already on treatment at programme entry and those diagnosed by pre-ART screening. Incident TB was any new episode of TB treatment after ART initiation.

Virological failure was defined as any HIV RNA >25 copies/ml, this definition was used not to indicate clinical treatment failure but to indicate those who have unsuppressed viral replication for this study. Lost to follow-up was defined as no clinic attendance on two consecutive occasions (2 months) and limited tracking of those defaulting treatment was available with telephone calls. No information was available on outcomes from the separate DoH TB programme.

## Results

**Figure 1:**
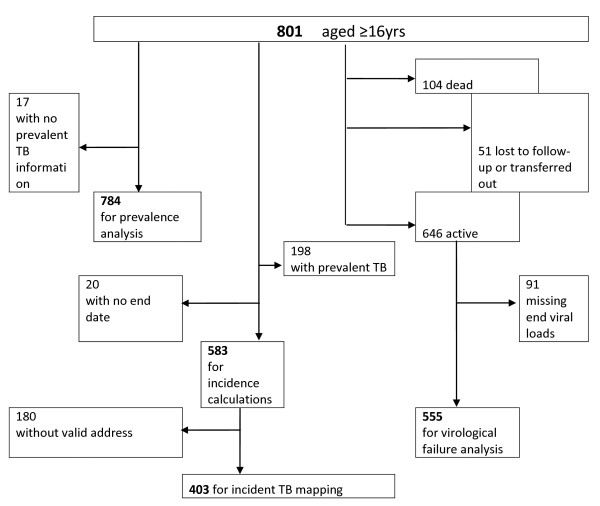
**Flow diagram illustrating patients used in the analysis**.

801 adults aged 16 or older started ART between the 1^st ^January 2005 and 31^st ^March 2006 (Figure 1) and had files for review (missing files n = 83). Those transferred in from another government programme were included. Transfers from GP or non-governmental programmes (n = 8) were included for TB prevalence analysis but removed before incidence and survival analysis because of unknown previous ART regimens. There were 258 missing baseline viral loads and 25 missing baseline CD4 counts.

Median age at treatment initiation was 36 years (IQR 31-44), 561 (69.9%) were female. 50.6% (n = 405) were WHO stage 3, 9.0% (n = 72) stage 4. WHO staging was not recorded for 98 (12.2%). Median CD4 count was 119.5 cells/μl (IQR 60-175) and median log_10 _HIV RNA 4.76 copies/ml (IQR 4.15-5.26).

### Prevalent TB

784 (of 801) had a complete TB record at ART initiation; 198 of whom (25.3%) had prevalent TB (95%CI 22.3-28.6%). Of those with prevalent TB, 84 had also had prior TB (42.9%). Prevalent TB was associated with CD4 below 50 cells/μl, higher baseline Log_10 _HIV RNA and prior TB (Table 1). No data was available on method of diagnosis in prevalent TB cases.

**Table 1:** Description of baseline characteristics stratified by prevalent TB and univariate and multivariate analysis of factors associated with prevalent TB.

Variable	Prevalent TB n(%)	No Prevalent TB n(%)	Unadjusted OR (95%CI)	Adjusted OR (95%CI)
**Sex**				
**Female**	136 (68.7)	413 (70.5)	REF	
**Male**	62 (31.3)	172 (29.5)	1.09 (0.77-1.54)	

**Age (yrs)**	35 (IQR 30-42)	37 (IQR 31-44)	0.98 (0.97-1.00)	0.99 (0.97-1.01)

**Transfer in**				
**No**	192 (97.0)	563 (96.1)	REF	
**Yes**	6 (3.0)	23 (3.9)	0.76 (0.31-1.91)	

**CD4 at initiation**	88 [IQR 30-153]	129 [IQR 71-179]		
**>200**			REF	REF
**50-200**			1.30 (0.80-2.10)	1.03 (0.58-1.86)
**<50**			3.22 (1.88-5.51)	2.61 (1.36-5.03)

**Baseline RNA VL (log_10_)**	4.91	4.70	1.26 (1.01-1.58)	1.23(0.99-1.54)

**Prior TB**				
**No**	113 (57.1)	387 (66.0)	REF	REF
**Yes**	85 (42.9)	199 (34.0)	1.46 (1.05-2.03)	1.58 (1.06-2.36)

**WHO Stage**				
**1 or 2**	N/A	214 (36.5)	N/A	N/A
**3 or 4**	198 (100)	282 (48.1)	N/A	N/A
**Unknown**	N/A	90 (15.4)	N/A	N/A

### Outcomes

The median follow-up time was 2.18 years (range 0-3.50). 646 (77%) were still active at the time of data censoring, 104 (13.0%) had died and 51 (6.4%) were lost to follow-up or transferred out.

Of the 46 lost to follow-up, median time to loss was 522 person days (IQR 223-706), median baseline CD4 137 cells/μl (IQR 60-162) and log_10 _HIV RNA 4.59 copies/ml (IQR 4.23-5.26), 37 had no prevalent or incident TB, 9 had prevalent and 6 incident TB.

### Incident TB

583 observations were used for analysis; 198 with prevalent TB were excluded and 20 others had missing end date leaving 81 episodes of TB analysed over 1175 years analysis time. Overall incidence was 6.89 per 100 person years, highest in the first three months at 17.4 per 100 person years (Table 2), and 5.33 per 100 person years after 2 years. In multivariate analysis young age, male sex and higher log_10 _HIV RNA (Table 3) were associated with incident TB in the first three months. After three months, advanced HIV stage (3 or 4) was significantly associated with incident TB. Prior TB was not independently associated with incident TB.

**Table 2:** Baseline characteristics associated with incident TB stratified by time since ART initiation

Time since ART initiation (months)	Person time (years)	TB cases	TB incidence rate (per 100 person years)	95% confidence interval
**0-3**	138.00	24	17.39	11.65-25.94
**3-6**	130.65	11	8.42	4.66-15.20
**6-9**	126.44	7	5.53	2.64-11.61
**9-12**	122.67	7	5.71	2.72-11.97
**12-18**	236.84	13	5.48	3.18-9.45
**18-24**	214.20	8	3.73	1.87-7.47
**>24**	206.34	11	5.33	2.95-9.63
**Overall**	1175.17	81	6.89	5.54-8.57

**Table 3:** Changes in TB incidence rate during antiretroviral therapy among 583 patients initiating treatment.

	Initial 3 months (n = 583 individuals)		After 3 months (n = 559 individuals)	
**Variable**	**Unadjusted Hazard Ratio (95% CI)**	**Adjusted Hazard Ratio (95% CI)**	**Unadjusted Hazard Ratio (95% CI)**	**Adjusted Hazard Ratio (95% CI)**

**Age (years)**	0.99 (0.95-1.04)	0.87 (0.79-0.96)	0.98 (0.95-1.10)	N/S

**Sex;**				
**Female**	REF		REF	
**Male**	4.17 (1.83-.54)	9.55 (2.35-38.78)	0.59 (0.30-1.16)	0.55 (0.27-1.10)

**Log_10 _HIV RNA**	1.85 (0.86-3.97)	2.69 (1.11-6.51)	0.83 (0.59-1.13)	N/S

**Transfer in**	0.99 (0.13-7.30)	N/S	0.38 (0.05-2.78)	N/S

**Log_10 _CD4**	1.35 (0.78-2.40)	2.02 (0.58-4.76)	0.83 (0.65-1.07)	0.87(0.67-1.13)

**Prior TB;**				
**No**	REF		REF	
**Yes**	1.42 (0.63-3.20)	1.16 (0.33-4.03)	1.77 (1.04-2.98)	1.32 (0.73-2.38)

**WHO stage**				
**1 or 2**	REF		REF	
**3 or 4**	2.33 (0.92-5.92)	N/S	2.11 (1.15-3.87)	1.94 (0.99-3.38)
**Unknown**	0.40 (0.05-3.32)	N/S	1.27 (0.52-3.12)	1.43 (0.58-3.56)

Of the above 81 individuals who developed TB whilst on ART, 14 (17.3%) were smear positive and 1 culture positive, 17 (20.1%) did not have the method of diagnosis recorded, 30 (37.0%) were diagnosed on CXR. 14 were diagnosed on clinical suspicion (17.3%), 1 on CT findings (1.2%), 2 on lumbar puncture findings (2.5%) and 2 on ultrasound findings.

### Spatial Clustering of Incident TB

**Figure 2:**
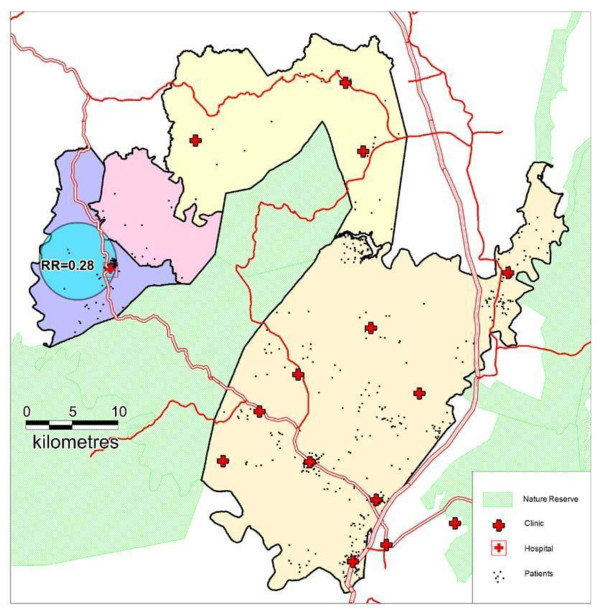
**Map of Hlabisa sub-district illustrating clinics and 403 patients with no prevalent TB at baseline**. The low-risk incident cluster around Hlabisa Hospital of radius = 4.2 km is shown.

403 individuals (821 person-years of observation) without prevalent TB and with a valid address were geocoded to 79 local areas. No clusters of unusually high TB incidence were detected anywhere in the sub-district. A significantly low-risk cluster of radius 4.2 km was detected in communities around Hlabisa Hospital (Figure 2). The overwhelming majority (95%) of person-years of observation in this cluster were contributed by patients geo-coded to the area immediately surrounding the hospital. In this cluster, only six new TB cases were observed over 151.9 person-years of observation - approximately one quarter of the expected incident cases (Relative Risk = 0.28, log likelihood ratio = 11.0, p = 0.019).

### Mortality

Total follow-up time was 1706 person years with an overall mortality of 6.3 per 100 person years (95% CI 5.2-7.6). 13 people had to be excluded from the outcomes analysis because of missing end date (i.e. no date of death or date of last follow-up available).

**Figure 3:**
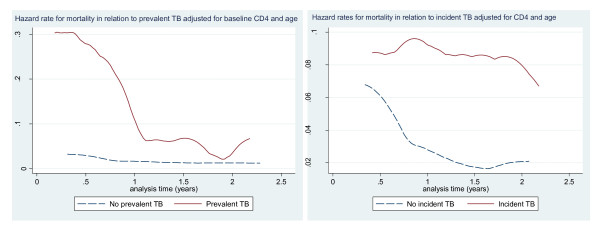
**Hazard rates illustrating mortality in relation to (a) prevalent TB and (b) incident TB**.

Mortality rate was significantly higher in those with prevalent and incident TB than in those without; 9.1 per 100 person years (95% CI 6.6-12.6) versus 5.3 (95% CI 4.2-6.8) and 9.2 (95% CI 5.6-15.0) versus 4.4 (95% CI 3.3-5.8) respectively. Similarly, mortality was 21.2% in incident TB cases, 11.8% in those without; 19% in prevalent TB, and 11.5% in those without (chi-2 p-value of 0.007, p = 0.008). Hazard risk for mortality in those with prevalent TB was 10 times higher in the first six months after initiation than later (Figure 3a), and was also higher in those with incident TB compared to those without incident TB (Figure 3b)

### Virological Outcome

Virological failure occurred in 71 (12.8%) of 555 people with follow-up viral loads. Median time from initiation to last viral load was 900 days (range 349-1336). 12 prevalent TB cases (9.4% of the 128 with prevalent TB) had virological failure versus 59 (14.4% of 411 without prevalent TB) p = 0.15. Conversely, virological failure was more common in incident TB cases than those without (12/63; 19.1% versus 58/490; 11.8%(p = 0.11)).

## Discussion

The extremely high TB prevalence in our study demonstrate that South Africa's dual HIV and TB challenge is not confined to urban settings; this rural cohort had a TB prevalence of 25.3%, similar to urban South Africa [8,17] and higher than figures reported elsewhere in sub-Saharan Africa [7,18,19].

HIV associated immunosuppression is known to increase the development of active TB in those latently infected and increase susceptibility to new TB infection [20,21]. We show that people with a CD4 count of less than 50 cells/μl prior to ART initiation were more likely to have prevalent TB, which is consistent with previous findings [8,19].

The incidence of TB following ART initiation was similar to that observed within an urban community in Cape Town [8]. Despite similar cohort characteristics, the incidence within the first 3 months in our study, whilst within confidence intervals, is slightly lower. In that study, sputum culture was part of the work-up, whilst in our setting culture was performed infrequently. Given that much of the TB is pulmonary, this again highlights the need for more active and aggressive diagnosis within a rural setting.

The high incidence of TB in the initial three months of ART compared with later may reflect an immune reconstitution inflammatory syndrome (IRIS) to TB infection which was present, yet undiagnosed at the time of ART initiation; this phenomenon has been termed unmasking tuberculosis-associated IRIS [22]. In this analysis, incidence refers to the incidence of diagnosis of disease rather than disease per se. The quoted figures for prevalence are therefore of prevalent diagnosed disease and the actual prevalence of disease might be higher. Incident calculations are not affected by high numbers of new TB diagnosis in the first two weeks, however, as this was only recorded in 4 cases; removal of these 4 from incidence calculations does not affect the final rate. Obviously it is impossible to comment on deceased or lost to follow-up cases.

The association between previous, prevalent and incident TB is complex and the subject of some debate [8,23] and conflicting evidence [8,19,24]. Our study is limited in the detail available about previous treatment and the number of follow-up CD4 results available; and therefore we were unable to confirm an association between higher CD4 attainment during ART and reduced TB incidence. However, we did find that previous TB was independently associated with prevalent TB at initiation, for which there are a number of possible explanations. In areas of high transmission, for example particular households or workplaces, re-infection may be common, particularly amongst the immunologically vulnerable. High re-infection rates may also be explained by nosocomial spread of TB within hospitals and clinics [25]. Delayed diagnoses coupled with lack of infection control adds to the increased risk of re-infection. Previous poor treatment adherence is another potential explanation, as relapse of partially treated disease as immunodeficiency advances could account for retreatment. In the absence of routine culture based testing it is not possible to tell whether drug resistance TB could account for the significant number of individuals requiring retreatment, but community rates of drug resistance are high with 40-55% [26] of individuals who gave culture specimens on retreatment showing evidence of some drug resistance, and 27% retreatment cases identified as MDR [27]. Drug resistant TB in the HIV setting is of particular concern for the control of both epidemics [6].

Separately analysing incidence in the initial three months and thereafter allows us to illustrate the factors strongly associated with incident TB. Male sex and younger age were not risk factors in the cohorts in Cape Town, Uganda and the Ivory Coast [8,19,24] but were in the ART-LINC cohort [28] and male sex, lower CD4 and lower BMI were associated with incident TB in Johannesburg [29]. Male gender has been historically linked to increased TB incidence [30] but in our study was associated with TB incidence only in the first three months post ART initiation. This may be attributable to illness behaviour; symptom denial at the time of presentation for ART initiation may be more common in men and therefore higher rates of immune reconstitution TB may be expected in men. Male sex in previous South African studies has been associated with increased delay in diagnosis of TB [31]. Work related or social behavioural exposure such as travel in minibus taxis may be more common in men, and these may be areas of target for active case finding activities for both TB and HIV, it would be expected however, that this higher rate in men continue after three months which it does not in this study. After the initial three months, we found that only advanced HIV stage was significantly associated with incident TB, a finding which supports other evidence that more advanced disease at initiation is associated with increased risk of TB disease [28].

The high TB incidence and high mortality in the first three months after initiation of ART necessitates improved active case finding through, amongst other things, optimised screening practices. The high mortality from TB in HIV infected individuals has been clearly illustrated in South Africa [32]. The challenge can best be addressed by the integration of TB and HIV services [33], especially in the most rural and under-resourced areas. Further, there is potential for investigating the use of targeted chemoprophylaxis with isoniazid possibly both pre and post ART initiation; although there remains significant concern about this approach in an area of high levels of TB drug resistance [34].

Previous studies have shown similar virological outcomes in HIV patients on ART taking concurrent TB treatment and those not taking TB treatment [8,35]. However, these were urban patients and follow up was less than two years. We have illustrated a non-significant trend towards detectable viral load in cases with incident TB. A longer period of follow-up might reveal significance. What is clear from our data is the strong association between prevalent and incident TB and mortality. This illustrates an opportunity to select groups in need of increased support and monitoring and advocates for the prevention of late presentation with illness on, and before ART.

Interestingly, the incidence of new TB cases was not geographically uniform across the sub-district and a cluster of unusually low TB incidence was detected surrounding the district hospital. These communities close to the hospital have easy access to diagnostic investigations such as chest x-rays, and were within easiest access of the first ART services in 2004/5. Both of these reasons may have contributed to earlier ART initiation in comparison to the rest of the sub-district. This in turn may have resulted in a reduced risk of incident TB, and earlier access to TB diagnosis and treatment leading to a reduction in the community's overall burden of TB disease.

However, 40% of patients geo-coded to the low-risk cluster reported living in the Hlabisa tribal area but only supplied a postal (and not a physical) address. It is therefore at least theoretically possible that a social rather than a geographical clustering pattern is being detected. This explanation is improbable, however, given the high population concentration in this area and the fact that individuals are likely to live close to the area where their post is collected. The use of geospatial techniques for analysing clustering of TB cases will aid understanding of the TB epidemic in rural sub-Saharan Africa (SSA), similar to the way in which mapping of HIV cases has highlighted not only areas where high transmission is occurring (along major transit routes [36]) but also the targeting of areas where infection control interventions will be most effective. This analysis is particularly important in HIV and TB co-infection which remains a leading killer in SSA.

Our study has several limitations. The original purpose of this programme is service and not research and care is largely provided by nurses and counsellors. It is therefore possible that the information recorded does not give a full picture of TB incidence. Further, in this sub-district of 2280,000 people, where over 3,500 TB cases are diagnosed annually, and as many as 80% of pulmonary cases are smear negative (Tom Heller, personal communication March 2009), the possibility of over-diagnosing TB on clinical grounds cannot be dismissed. We are restricted by lack of information on the cause of mortality or the status of lost to follow-up patients. It is possible that several of our deceased patients had incident TB, and some of the defaulted patients may have been started on TB treatment.

In our study, the absence of data from the era prior to ART hinders drawing conclusions about the potential impact of ART on the risk of developing TB. Studies in urban South Africa suggest a reduced incidence of TB for HIV infected people taking ART before and after national roll-out [8,37] and some have suggested that the reduction in TB incidence in high and low-income countries after HAART is as high as 70-90% [38]. However there is lack of evidence for rural populations which face different challenges. The risk of TB whilst on ART during long term follow-up becomes stable in our setting at a rate six times higher than the overall TB incidence in KwaZulu-Natal [4] which supports suggestions that ART alone will not be a fully effective TB control strategy for several reasons, including the lack of restoration of MTB immune response on ART to that of an HIV negative person coupled with prolonged lifespan after ART [38].

## Conclusion

To impact on the high rates of both prevalent and incident TB in this and other resource poor settings, and to prevent associated high mortality and morbidity, a combination of strategies must be used. This should include preventing late entry into HIV care and onto ART, infection control measures around TB, targeted enhanced screening and enhanced adherence support for those on both TB and HIV treatment. The data presented here and reduced incidence of TB around the hospital also suggests that more emphasis should be placed on strengthening services away from the hospital, either in primary care or at a household level. In order for these strategies to be as sustainable and as effective as possible, they will need to be integrated with each other and into existing primary health care for other chronic diseases. Further understanding of the TB epidemic in HIV infected individuals will be greatly enhanced by the use of geospatial analysis of cases. The development of TB resistance remains an important factor driving the ongoing TB epidemic, a worse tragedy will be if TB further encourages HIV resistance and prevents ART roll-out from impacting on the high mortality rates.

## Competing interests

The authors declare that they have no competing interests.

## Authors' contributions

CH contributed to study design, data collection, data analysis and writing of the manuscript. PM contributed to study design, data analysis and writing of the manuscript, GC contributed to study design and writing of the manuscript, RL contributed to study design and writing of the manuscript, FT contributed to data analysis and writing of the manuscript and performed the clustering analysis, MN contributed to study design, data analysis and writing of the manuscript. All authors have read and approved the final manuscript.

## Pre-publication history

The pre-publication history for this paper can be accessed here:

http://www.biomedcentral.com/1471-2334/10/23/prepub
